# Management of Polytraumatized Patients: Challenges and Insights into Air Transfer

**DOI:** 10.3390/healthcare13172181

**Published:** 2025-09-01

**Authors:** Mihaela Anghele, Cosmina-Alina Moscu, Liliana Dragomir, Alina-Maria Lescai, Aurelian-Dumitrache Anghele, Alexia Anastasia Ștefania Baltă

**Affiliations:** 1 Faculty of Medicine and Pharmacy, “Dunărea de Jos” University of Galați, 800008 Galați, Romania; anghele.mihaela@yahoo.com (M.A.); lilianadragomir2017@gmail.com (L.D.); anghele_aurelian@yahoo.com (A.-D.A.); alexiabalta@yahoo.ro (A.A.Ș.B.); 2 “St. Apostle Andrei” Clinical Emergency County Hospital, 800578 Galați, Romania

**Keywords:** polytrauma patient, car crash polytrauma, more than three regions affected by polytrauma

## Abstract

Background and Objectives: Despite the potential benefits for the patient, aerospace interventions pose significant risks. Pre-hospital triage and patient transport are two essential elements for achieving an organized system of trauma. The advantages and disadvantages of using land transport from the scene of the accident to the trauma centers have been extensively studied, but there are gaps for air transport, and their exact level of efficiency is not known. Materials and Methods: The present study includes a total number of 77 patients, present at SMURD Galați air service for polytraumas caused by various mechanisms, with pluri-regional involvement. The identification of patients, as well as the selection of the most important anamnestic data, was performed after signing a confidentiality agreement; subsequently, all this information was introduced in centralized tables made in the statistical program IBM SPSS Statistics V24. Results: Out of the total of 77 polytraumatized patients who needed air transfer, an average age of 17.3 years will be noted, with a predominance of males in a 2:1 ratio. Most polytraumas are due to road accidents (74%) and patients with minimal tri-regional damage (51.4%). Conclusions: Taking into account the existing statistics in this research, it is important to implement prevention elements, designed based on the profile of the polytraumatized patient. Thus, accessing the most important characteristics of these patients can be an extremely important starting point in reducing the incidence of polytrauma or even patient deaths.

## 1. Introduction

Air ambulances are an integrated component of modern care for traumatic pathologies. They are able to transport patients to facilities with higher capacities, to extract injured patients from hostile terrain, and to transport them quickly to a trauma center [1].

Pre-hospital triage and patient transport are two essential elements for achieving an organized system of trauma. Current studies do not clearly define the benefits of helicopter transport over mortality in a prehospital trauma system with medical staff [2].

Recent research has provided evidence supporting broader assumptions about air transport efficiency. This demonstrates that air transport does not reduce the total time required to transport a patient, but reduces the time spent by the patient between medical units [1]. As a general conclusion, these studies demonstrated the importance of factors with the potential to influence patients’ conditions. Key influencing factors include the availability of advanced interventions, qualified staff, prompt service, and access to specialized trauma centers [3].

While air medical transport offers notable clinical advantages, it is also associated with distinct operational risks. Adverse weather conditions, limited visibility, and night-time operations can compromise both safety and mission effectiveness. Consequently, the decision to deploy air ambulances must be guided by a balanced consideration of clinical urgency and operational safety constraints [4,5].

Although air transport may not significantly reduce the total transport time, it plays a crucial role in shortening the time required to access specialized trauma centers, especially during inter-facility transfers. Also, recent studies have shown that the presence of a doctor during helicopter transfer improves patient outcomes [5,6,7].

For this reason, although the air transport of patients has been widely available for several decades, it is still used with great caution (the benefits of this still require more extensive studies).

The benefits associated with air transport by helicopter are influenced by many variables specific to each mission and include the severity of injuries, their mechanism of production, the ability to transport patients from hard to reach or long distances, and specific transport algorithms [4]. While land transport has been widely studied, the efficiency of air transport in trauma cases remains uncertain and under-researched [6,8].

Since 1997, Mobile Emergency Service for Resuscitation and Extrication (S.M.U.R.D.), an institution that is intended to rescue critically ill patients, has been carrying out emergency aero-medical interventions and, in Romania, air rescue activity has been carried out by it since 2004 [9].

This service is a public one, regulated by the Government Emergency Ordinance no. 126/2003 on the operation, functioning, and financing of emergency assistance and was provided with helicopters purchased by the Ministry of Health and distributed to medical operators Fundeni Clinical Institute and Targu Mures County Emergency Clinical Hospital, by order of the Minister of Administration and Interior and the Minister of Health no. 277/777/2004 for the approval of the methodological norms for the application of GEO no. 126/2003 [9].

S.M.U.R.D. rescue missions are carried out with the light multifunctional Eurocopter EC 135 helicopter, equipped with the Special Aviation Unit of the Ministry of Internal Affairs and Administrative Reform. In the context of the Romanian S.M.U.R.D. aeromedical service, primary missions are direct interventions at the scene of an accident in order to stabilize and transport the patient to a medical center, while secondary missions involve the inter-clinical transfer of critical patients between health facilities in order to rapidly access specialized or higher level medical services [9].

Research on patient air transport has been limited to specific pathologies, so no general guideline has been developed to facilitate the choice of the most appropriate mode of transport, nor the definition of over-triage when the helicopter is used to perform those inter-clinical transfers [6,8].

Considering the local specificity and the available resources, the aim of this study is to characterize the clinical and demographic profile of polytraumatized patients transported by air by the S.M.U.R.D. Galați service, as well as to analyze the main interventions applied during the transfer. The study aims to provide descriptive data useful for improving the organization of aeromedical transport services, especially in regions with limited access to trauma centers.

Although a direct comparison with land transport cannot be made due to the lack of a control group, we hypothesize that air transfer provides operational and logistical benefits in accessing specialized care for polytrauma cases, particularly in geographically isolated areas. However, due to the lack of complete and standardized data on transport duration and clinical outcomes (e.g., survival and hospitalization length), our analysis is limited to clinical and operational aspects observed during the transfer.

## 2. Material and Methods

**Figure 1:**
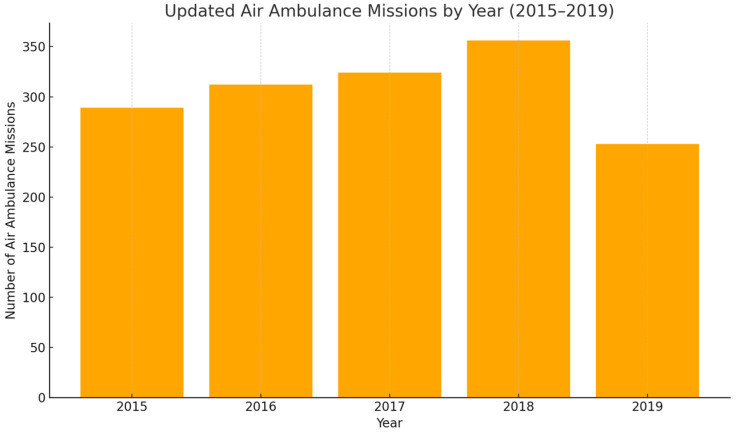
Update of air ambulance missions by year (2015–2019).

The present study includes a database consisting of a total of 77 patients (young adults and pediatric patients), known to have multiple injuries through various mechanisms of production that required transport by air ambulance. The study is retrospective, as the information about the subjects was collected over 5 years, between 2015 and 2019. During the analyzed period, the SMURD Galați air ambulance service conducted a total of 1534 missions, distributed annually in a relatively balanced manner, with a peak in 2018 and a slight decrease in 2019, as illustrated in Figure 1.

The inclusion criteria were patients with multiregional polytrauma, transported by the S.M.U.R.D. Galați air service between 2015 and 2019, and with complete medical data available. Exclusion criteria were as follows: patients with incomplete data, patients transported terrestrially, or cases with isolated trauma not requiring complex management. Parameters analyzed included demographics (age, sex), mechanism of trauma, Glasgow Coma Score (GCS), respiratory rate, ventricular rate, oxygen saturation (SpO_2_, interventions applied (oxygen administration, artificial ventilation, intubation, RSI, cardiac massage), location of injuries, number of regions affected and type of mission (primary, secondary). Data were sorted and filtered according to clinically and statistically relevant criteria, but no information was collected on transport duration, survival, or length of hospitalization, which are proposed for future prospective research.

The data obtained from the research of the patient observation sheets were introduced in the statistical analysis program (IBM Statistics V. 24 *SPSS, Inc., Chicago, IL, USA) and Excel 2019, subsequently being filtered and sorted according to different criteria. Initially, the gross descriptive statistical parameters were calculated for all the variables for which this type of calculation approach was considered potentially useful. These include mean value, standard deviation (SD), minimum and maximum value for continuous numerical variables, frequency for categorical ones, median and mode value, and skewness and kurtosis indices. From a descriptive point of view, the corresponding diagrams were used for the graphical representations, using the software applications dedicated to the programs.

Categorical values were entered in the contingency tables and the non-parametric chi-square test (χ^2^) was applied. For the calculation of the central trend and data dispersion, descriptive statistics were used, using the 95% confidence interval (confidence interval, 95%), the standard error of the mean, and the minimum and maximum value. For each of the existing statistical tests, a level of statistical significance for values of 0.05 was used, being calculated by the value of the *p*-index at two ends. Student’s t-test was used to highlight the statistically significant differences between the groups or subgroups generated within the study group.

No information was collected on transport duration, time from incident to arrival of medical personnel, survival, or hospitalization length. These limitations are due to inconsistent or missing documentation in retrospective records and are acknowledged as critical aspects to be addressed in future research.

## 3. Results

The character of the missions of the SMURD crew is as follows: in a proportion of 59.7%, the mission has a primary character, followed by the missions with secondary character (29 patients, 37.7%). The smallest weights are represented in a proportion of 2.6% by special/rescue missions.

This section presents the findings of an analysis of 77 polytrauma patients transported by air ambulance. These findings are derived from a detailed examination of patient demographics, injury mechanisms, vital parameters, and statistical correlations, offering insights into the challenges and complexities of managing polytrauma cases.

### 3.1. Demographics and Injury Mechanisms

**Figure 2:**
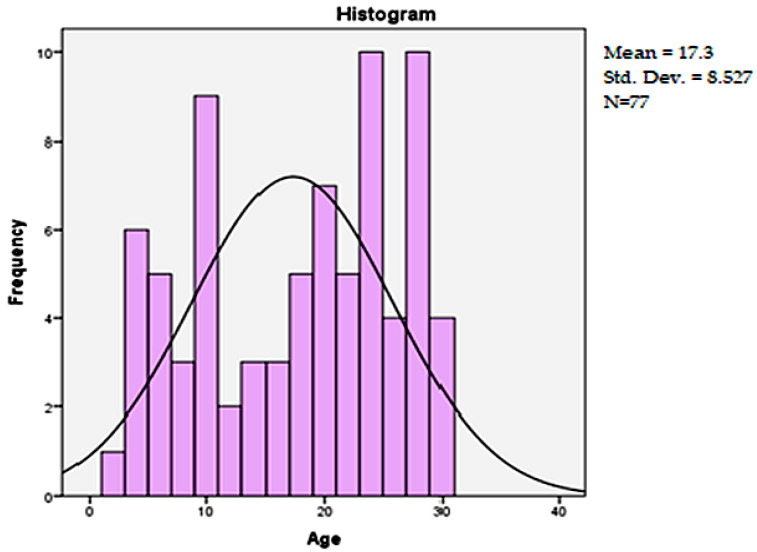
Age distribution of polytrauma patients transported by air ambulance (*n* = 77).

Out of the 77 patients, the majority were male (66.2%, *n* = 51), with a male-to-female ratio of 2:1. Figure 2 shows the distribution of patient ages. The average age was 17.3 years (±8.52), with notable peaks around 10 and 25 years. Most injuries (74%) were caused by road traffic accidents, encompassing drivers, passengers, and pedestrians. Additionally, 13% of injuries resulted from falls, while 11.7% were attributed to mechanisms such as crushing or physical aggression.

### 3.2. Patterns of Injury and Severity

**Figure 3:**
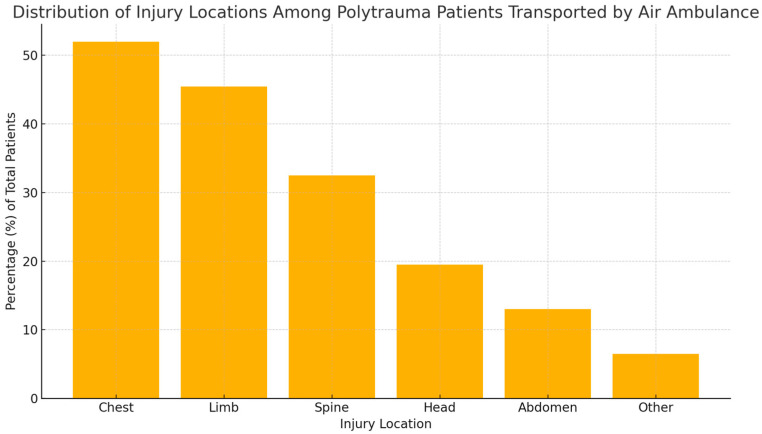
Distribution of injury locations among polytrauma patients transported by air ambulance.

Injury localization analysis showed that 51.9% of patients presented with tri-regional trauma, with a predominance of thoracic injuries (52%), followed by injuries to the limbs (45.5%) and spine (32.5%). Head injuries were reported in 19.5% of patients, abdominal injuries in 13%, and other injuries accounted for 6.5%. The percentage distribution of these categories is illustrated in Figure 3, where the vertical axis expresses the proportion (%) of the total 77 patients included in the study.

### 3.3. Clinical Parameters and Interventions

The main clinical parameters are summarized in Table 1.

The results of the Glasgow Coma Scale (GCS) scores ranged from 3 to 15, with an average value of 10.82 and a standard deviation of 5.25. The values of skewness and kurtosis indicate a homogeneous distribution. Respiratory rate analysis showed an average value of 9.91 breaths per minute, with a standard deviation of 7.499 breaths per minute. The ventricular rate exhibited a maximum value of 161 bpm, with an average of 95.60 bpm and a standard deviation of 35.09 bpm.

Oxygen saturation levels ranged widely, with a mean value of 90.95% and a maximum of 100%. The highest values were recorded after medical interventions during air transfer. Upon arrival at the scene or during initial evaluation, several patients presented with moderate to severe hypoxemia. Consequently, oxygen supplementation was required in 68.8% of cases, most frequently via simple mask or assisted ventilation, to stabilize respiratory function before or during transfer.

Among the 77 patients included in the study, 12 individuals (15.6%) were identified as hypotensive at the time of initial assessment, defined by a systolic blood pressure below 90 mmHg. All hypotensive patients required oxygen supplementation, while 75% (*n* = 9) underwent rapid sequence induction (RSI) for airway management. Endotracheal intubation was successfully performed in six of these cases. All hypotensive patients received intravenous crystalloid fluids, and vasopressor therapy was initiated in three cases to manage persistent hypotension. No patients received blood products, tranexamic acid (TXA), fibrinogen, or invasive monitoring, reflecting the logistical limitations and emergency protocols applicable during air transfer. Intravenous fluid resuscitation with crystalloids was administered in all 12 patients. Additionally, vasopressor therapy was initiated in three cases to manage persistent hypotension. This subgroup exhibited significantly lower Glasgow Coma Scale (GCS) scores, necessitating immediate stabilization measures during air transport.

Of the total group analyzed, only 3 subjects presented cardiac arrhythmias: 2 patients (2.6%) experienced tachycardia, while 1 patient (1.3%) experienced asystole. Additionally, one patient (1.3%) presented with severe mixed dyspnea.

Of the total patients analyzed, 68.8% required oxygen administration during air transfer, of which, 35.1% received oxygen by simple mask. A total of 19.5% required artificial ventilation, defined in this context as assisted ventilation by devices such as bag-valve-mask (BVM) in the absence of intubation. Orotracheal intubation was performed in 6.5% of patients, while the Rapid Sequence Intubation (RSI) protocol was initiated in 20.8% of patients. However, orotracheal intubation was completed in only 6.5% of cases due to various clinical reasons, including patient instability, spontaneous recovery of respiration, or constraints related to the limited duration of transfer. No supraglottic airway devices (such as laryngeal mask airways) were recorded in the documentation, which may reflect equipment availability or crew preference during the analyzed missions. It should be noted that RSI was applied as a preparatory measure for intubation by administering sedatives and muscle relaxants, but not all of these cases were completed with endotracheal intubation due to various clinical reasons (instability, spontaneous breathing recovery, or constraints related to the duration of transfer). Also, 2.6% of patients required external cardiac massage.

### 3.4. Statistical Correlations and Observations

**Figure 4:**
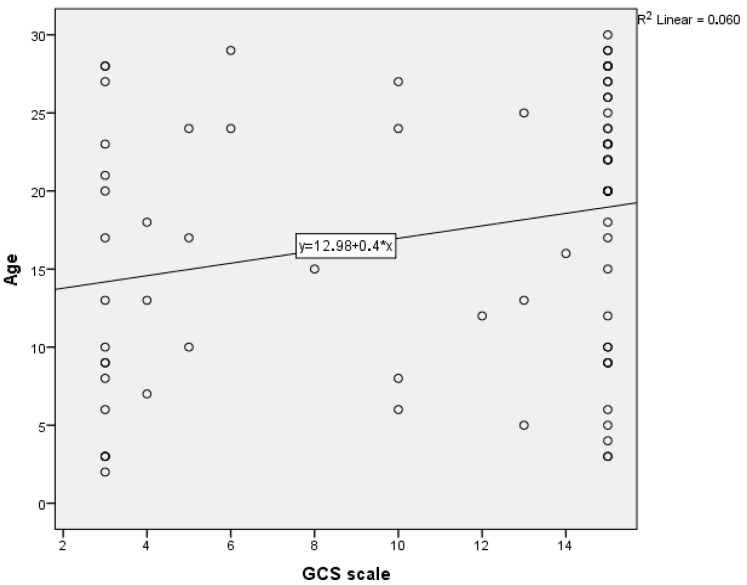
Correlation between Glasgow Coma Scale (GCS) and age.

Statistical analyses revealed significant relationships between two scalar variables: age (in years) and GCS scores. Figure 4 illustrates the graphical representation of the linear regression analysis between these variables, which provides the potential to make predictions. The regression equation indicates a weak positive correlation (R^2^ = 0.060), meaning that only a small portion of GCS variation is explained by age. This suggests that only 6% of the variation in GCS scores can be attributed to age, highlighting a limited dependency. The scatter plot shows variability, particularly among younger patients with lower GCS scores, reflecting the severity of their neurological impairment.

In addition, Table 2 displays the results of a paired-samples t-test conducted to evaluate the differences between the two variables. The test revealed a statistically significant mean difference of 6.481 (*p* < 0.001) between age and GCS scores, with a 95% confidence interval ranging from 4.472 to 8.489. These findings emphasize the systematic disparity between the two variables, demonstrating that younger patients are more likely to have lower GCS scores, indicative of greater trauma severity.

In the following, we tried to find statistically significant correlations to highlight the relationships of dependence or even causality between individual variables. Thus, the first intention ANOVA testing was performed to quantify the degree of dependence of patients’ age (as a scalar variable, dependent) in relation to the mechanisms of production or the number of affected regions. Thus, it was observed that between age and the mode of production of polytrauma there is no statistically significant correlation according to the ANOVA test (Levene = 0.410, ANOVA = 0.452). The same situation is encountered in the case of the interaction of this scalar variable in relation to the number of affected regions, no statistically significant correlation being demonstrated (Levene = 0.986, sig. ANOVA = 0.679).

Pearson index bivariate correlations were used for continuous variables. They did not detect any statistically significant correlations except for those between the mechanism of polytrauma production and the need for oro-tracheal intubation (sig = 0.038) as shown in Table 3.

### 3.5. Comparative Outcomes by Injury Mechanism

The information presented above (Table 4) is analyzed below, by descriptive statistics, by performing independent t-tests. Thus, two groups of patients were created by reference to the mechanism of polytrauma production, as follows: those who were victims of road accidents (57 patients of sublot A), respectively, and polytraumas due to other causes (20 patients of sublot B). The t-test results revealed significant differences, particularly regarding intubation needs and average age between subgroups A and B.

The GCS score (average age for polytraumas due to road accidents was 10.39 points, respectively, 12.05 points for the other sublot). Although the significant value according to the t-test of the equivalence of the averages is 0.225, it can be concluded that at the level of the group there were lower GCS scores in the case of polytraumas from sublot A.

There was a lack of statistically significant differences between the two sublots, in relation to the need for oxygen (sig = 0.832) or performing cardiac massage to stabilize the patient during the transfer (sig = 0.403).

In the case of intubations performed during the transfer, the existence of significant differences between the two sublots can be observed (sig of the Levene test less than 0.01, respectively, sig of the t-test of the equivalence of the averages of 0.01). For this reason, we reject the null hypothesis and admit that, in the case of the group analyzed in the present research, there are differences between sublots A and B in terms of trachial intubation and average age.

In Table 5 is presented a descriptive statistic of the different variables analyzed in this research, depending on the mechanisms of polytome production, being associated with the significant values according to the Chi square tests. The only variables between which significant differences were defined (sig = 0.000) are represented by the production mechanisms and the location of the affectation. Thus, it is noted that, in road accidents, the association of regions is present, while in polytraumas by fall the cephalic extremity is predominantly affected, and in polytraumas by other mechanisms, thoracic injuries is predominant.

## 4. Discussions

The results of this study provide detailed insights into the characteristics of polytrauma patients transported by air, as well as the interventions applied. When compared to the literature, the findings align with international observations, highlighting the challenges and benefits of this type of transport.

### 4.1. Demographic Profile and Trauma Mechanisms

Of the 77 patients analyzed, 66.2% were male, with a mean age of 17.3 years. This result is consistent with other studies that identify young males as the group at highest risk of severe polytrauma, especially due to their involvement in risky behaviors, such as aggressive driving or playing dangerous sports [10,11].

The distribution of trauma mechanisms shows that road traffic accidents were the main cause (74%), followed by falls (13%) and other mechanisms, such as physical assaults (11.7%).

These patterns align with established findings in the literature, indicating that high-energy trauma mechanisms, such as road traffic accidents, often result in injuries to the thoracic and extremity regions. Such injuries are frequently associated with significant morbidity and necessitate prompt and specialized medical intervention.

Although the database included both pediatric and adult patients, no statistical comparison was conducted between the two subgroups regarding injury mechanisms, injury types, or number of affected regions, due to the relatively small sample size and the skewed age distribution (mean age 17.3 years). This is acknowledged as a limitation.

These data are supported by Roshanaei et al. (2022), who identified road traffic accidents (39.6%) and falls (30.2%) as the predominant mechanisms of trauma among patients [12].

Similarly, Bradshaw et al. (2017) found that road traffic accidents and falls each accounted for 41% of pediatric trauma cases, highlighting variations between low- and high-income countries [13].

In contrast, in rural or mountainous areas, falls dominate as the main mechanism of injury, highlighting the importance of regional factors in determining trauma patterns [14].

Hulme (2015) supports this observation, noting that road traffic accidents and falls are the leading causes of death among children and adolescents in low- and middle-income countries [15].

These data also reflect the findings of Pascual-Marrero et al. (2018), who showed that road traffic accidents and falls are among the most common mechanisms of trauma, with a significant impact on morbidity and mortality in Puerto Rico [16].

In recent years, the effectiveness and outcomes of helicopter emergency medical services (HEMS) have been increasingly studied, particularly in trauma populations. For instance, Wejnarski et al. analyzed data from over 3000 patients with trauma or myocardial infarction transported by HEMS in Poland and reported significant differences in patient characteristics and types of missions compared to ground transport, underlining the strategic role of air transport in critical scenarios [17].

Similarly, Weinlich et al. evaluated over 1700 HEMS trauma cases in Germany and emphasized the added value of early intervention, advanced procedures, and shorter prehospital times, contributing to improved outcomes [18].

Our study shares similarities with these reports in terms of the predominance of trauma from road traffic accidents and the necessity for interventions such as oxygen administration and airway management. However, unlike these larger datasets, our cohort included a younger population (mean age 17.3 years), and procedures were adapted to the regional operational limitations and patient status during short transfers.

Moreover, Pham et al. found that faster on-scene times during HEMS missions were associated with reduced mortality in trauma patients, underscoring the critical importance of efficient scene management [19]. Although our study did not analyze scene times or patient outcomes, this highlights an important area for future prospective research.

Additionally, Lapidus et al. compared HEMS and road ambulance transports in Sweden and demonstrated superior survival rates in patients transported by helicopter, especially in rural or remote settings. These results suggest a potential benefit that may apply to systems like Romania’s, particularly in regions with long distances to trauma centers [20].

Taken together, these studies support the growing consensus that HEMS offers a meaningful advantage in selected trauma cases. While our findings are primarily descriptive, they align with international trends and further emphasize the need to standardize data collection, incorporate outcome measures, and facilitate inter-center comparisons in future research.

### 4.2. Location and Severity of Injuries

The majority of patients in this study presented with tri-regional injuries (51.9%), with a predominance of thoracic, extremity, and spinal injuries. These findings align with studies by Chilvers et al. (2017), who demonstrated that high-energy trauma most frequently affects these regions, especially in road traffic accidents [21].

Multiple injuries carry a significant risk of complications, including multiple organ failure. Studies by Biewener et al. (2004) confirm that combined chest and extremity injuries are associated with higher rates of intraoperative and postoperative complications [5]. The findings of this study further support the need for rigorous protocols for the rapid assessment and intervention of patients with severe polytrauma.

### 4.3. Clinical Parameters and Emergency Interventions

Clinical parameters indicate moderate severity of trauma, with a mean Glasgow Coma Scale (GCS) score of 10.82. In the literature, patients with GCS scores below 10 are considered to be at high risk of mortality, underscoring the urgency of rapid interventions for these cases [22]. Studies suggest that lower GCS scores are associated with an increased likelihood of intubation and poorer outcomes, especially in patients with severe trauma [23]. Emergency interventions included oxygen administration (68.8%) and orotracheal intubation (20.8%). It should be emphasized that the reported 100% SpO_2_ values reflect post-intervention measurements, not baseline saturation upon team arrival, thus supporting the necessity for oxygen therapy in a significant proportion of patients. Furthermore, the presence of hypotension in 15.6% of cases highlights the hemodynamic instability frequently encountered in polytrauma patients and reinforces the necessity for prompt and aggressive prehospital resuscitation strategies. These include early intravenous fluid administration, implementation of rapid sequence induction protocols, and definitive airway management to prevent further clinical deterioration during transfer. These data are comparable to those reported by Hoffmann et al. (2017), who highlighted that prehospital intubation was performed predominantly in patients with GCS ≤ 8, improving outcomes when combined with sedation [22]. Furthermore, McMullan et al. (2013) discussed the prevalence of prehospital oxygen use in trauma care, noting its critical role in cases of hypoxemia and hemorrhagic shock [24].

Patients with trauma also face a substantially elevated risk of venous thromboembolism, primarily due to prolonged immobilization and the physiological changes associated with severe injuries, as highlighted by Anghele et al. (2024) [25,26]. This underscores the need for vigilant monitoring and preventive strategies during both transport and hospitalization [25]. Additionally, the first 60 min post-trauma, often referred to as the “golden hour,” are crucial for determining patient survival and long-term outcomes [26].

Significant psychological challenges, such as intrusive thoughts and stress, are also common in polytrauma patients, which can delay recovery even in cases of successful medical interventions [27]. These findings emphasize the need for an integrated approach that addresses both physical and psychological factors in trauma care [28]. Furthermore, significant differences between patients involved in motor vehicle crashes and those with injuries from other mechanisms highlight the importance of personalizing intervention protocols, given the variable severity of injuries [29].

### 4.4. Impact of the Medical Team on Prognosis

Our study highlights the constant presence of a doctor and a nurse in all air transfers, in contrast to other research. Dewhurst et al. (2001) demonstrated that the presence of qualified medical personnel, such as anesthesiologists, is essential for the management of high-risk patients, reducing the incidence of adverse events, which were reported in 12% of cases [30].

Recent studies highlight the essential role of air transportation in emergency medicine, emphasizing its potential to reduce time to specialized care and improve patient outcomes. However, our study did not evaluate transfer times, and these references are cited here only to provide context. The presence of specialized medical personnel on board, particularly in severe cases, further contributes to the optimization of clinical management, leading to a reduced risk of complications and short-term mortality [31,32].

However, the prolonged exposure of healthcare professionals, particularly those in emergency departments, to high-stress environments significantly impacts their mental health and performance, as noted by Moscu et al. (2023), emphasizing the need for targeted support systems to mitigate these effect [33]. Mulrooney (1991) further highlighted the importance of physicians adapting to the unique conditions of the air environment to maintain or improve the condition of patients during transfers [34]. Moreover, the concept of the mobile intensive care unit (MOBI), introduced by Icenogle et al. (1988), demonstrated that the transport of critically ill patients with comprehensive medical care could lead to positive outcomes without complications during long-distance transfers, underscoring the crucial role of a well-equipped and adequately staffed medical team [35].

### 4.5. Implications for National Trauma Treatment Policy

The findings of this study can significantly support the development of clearer national policies for managing polytrauma in Romania. By outlining a detailed demographic and clinical profile of patients transported by air, the research provides a valuable framework for public health authorities and emergency response policymakers.

In particular, identifying the predominance of road traffic accidents and multiregional injuries justifies the need to revise prehospital triage criteria for air transport, ensuring that helicopter use is based on clearly defined clinical indications and resource efficiency. This may help reduce unnecessary over-triage and better allocate costly aeromedical resources.

Furthermore, correlations between GCS scores and patient age could inform updates to intervention algorithms, particularly for pediatric trauma cases. The study also underlines the need to establish a national trauma registry, incorporating structured aeromedical data to enable real-time protocol monitoring and optimization.

These conclusions may guide the strategic development of standardized national protocols for air transfer, improved training programs for aeromedical teams, and targeted investments in regional heliport infrastructure to enhance trauma system responsiveness. International studies also support this need; Tsuchiya et al. (2016) showed improved survival in trauma patients transported by helicopter, reinforcing the urgency of developing structured criteria for air transport use [36].

### 4.6. Comparison with Other Air Transport Systems

Compared to international systems, such as the United Kingdom, which reported over 221,000 emergency air missions between 1987 and 2009 [1], the Romanian system has limited resources. However, recent initiatives, such as the heliport at the Bucharest University Emergency Hospital, have demonstrated increased efficiency, with over 550 lives saved in five years [37]. These examples highlight the importance of investing in infrastructure and expanding capacity to meet international standards.

### 4.7. Limitations and Future Prospects

This study has several significant limitations that should be considered when interpreting the results.

The relatively small sample size of 77 patients limits the robustness of the conclusions and their applicability to national or international settings. In addition, the collection of data from a single geographic region and the retrospective study design may introduce biases, such as reliance on accurate medical documentation and selection of patients for air transfer, which could overestimate the benefits or challenges of this type of transport.

A key limitation is the absence of a control group consisting of polytrauma patients transported by land. Although this restricts the ability to draw direct comparative conclusions, our hypothesis remains that air transfer provides significant clinical and operational benefits in managing complex trauma cases, especially in situations where land access is limited or delayed. In such contexts, air transport ensures faster access to specialized trauma care, potentially improving patient outcomes despite the inherent risks and logistical challenges.

Additionally, the study population consisted predominantly of young patients, which reflects the real-world demographic profile of air-transferred trauma cases in the analyzed region but limits the generalizability of findings to other age groups.

A major limitation of this study is the absence of data on time-sensitive variables such as duration of transport and time from accident to initial medical contact, as well as outcome measures such as survival and length of hospitalization. These factors are essential for assessing the true impact of air medical transport and should be included in prospective, multicentric studies.

Furthermore, important variables such as long-term outcomes, operational conditions, or quality of life parameters were not included in the analysis. Future research should adopt a prospective design, include larger samples from multiple regions, and integrate control groups for a detailed comparison between air and ground transfers, with the aim of developing standardized national guidelines and assessing the impact on clinical outcomes and patient quality of life.

## 5. Conclusions

The study confirms the complexity of air-transferred polytrauma cases and describes the demographic and clinical profile of patients transported by helicopter in a regional Romanian context. The predominance of male patients and mechanisms such as road traffic accidents highlight the vulnerability of younger populations involved in high-risk activities. Tri-regional injuries and moderate severity of trauma reflect the need for coordinated prehospital care. Interventions such as oxygen administration, assisted ventilation, and intubation in selected cases illustrate the critical role of appropriately trained medical teams in the air transfer setting. Although the study did not evaluate outcomes or transfer duration, these descriptive findings can contribute to future discussions on optimizing aeromedical response protocols for trauma patients.

## Figures and Tables

**Table 1:** Descriptive statistics of clinical parameters.

	N	Range	Minimum	Maximum	Mean	Std. Deviation	Skewness		Kurtosis	
							Statistic	Std. Error (Skew.)	Statistic	Std. Error (Kurt.)
GCS Scale	77	12	3	15	10.82	5.253	−0.631	0.274	−1.485	0.541
Respiratory Rate	77	20	0	20	9.91	7.499	−0.387	0.274	−1.498	0.541
Ventricular Rate	77	161	0	161	95.6	35.09	−0.961	0.274	1.666	0.541
SpO_2_ (Oxygen Saturation)	77	100	0	100	90.95	24.479	−3.437	0.274	10.455	0.541
Systolic BP (mmHg)	77	120	70	120	108.7	22.6	−0.45	0.274	−0.28	0.541

**Table 2:** Paired *t*-test results comparing age and GCS.

	Mean	Std. Deviation	Std. Error Mean	95% Confidence Interval (Lower)	95% Confidence Interval (Upper)	T	Df	Sig. (2-Tailed)
Age–GCS Scale	6.481	8.848	1.008	4.472	8.489	6.427	76	0

**Table 3:** Pearson correlation between polytrauma variables and emergency response measures.

	Mechanism of the Polytrauma	Number of Regions Affected	Localization of the Polytrauma	Arrhythmias	Dyspnea	Oxygen	Intubation	External Cardiac Massage	Extrication	Restraint
Mechanism of the polytrauma	1	0.028	−0.127	0.216	−0.062	0.001	−0.237 *	−0.088	0.189	−0.207
Number of regions affected	0.028	1	0.552 **	0.093	0.152	0.12	0.131	0.11	−0.001	0.34
Localization of the polytrauma	−0.127	0.552 **	1	0.075	0.098	0.051	0.181	0.085	0.03	0.087

* Correlation is significant at the 0.05 level (2-tailed). ** Correlation is significant at the 0.01 level (2-tailed).

**Table 4:** Independent samples test.

Variable	Levene’s Test F	Sig.	T	Df	Sig. (2-Tailed)	Mean Difference	Std. Error Difference	95% CI Lower	95% CI Upper
GCS scale	0.543	0.464	−1.223	75	0.225	−1.664	1.361	−4.375	1.047
			−1.221	33.208	0.231	−1.664	1.363	−4.436	1.107
Oxygen	0.15	0.699	−0.213	75	0.832	−0.074	0.346	−1.736	1.588
			−0.211	32.661	0.834	−0.074	0.35	−0.786	0.638
Intubation	19.91	0	1.974	75	0.052	0.286	0.145	−0.003	0.575
			2.67	66.113	0.01	0.286	0.107	0.072	0.5
External cardiac massage	3.051	0.085	0.842	75	0.403	0.035	0.042	−0.048	0.118
			1.427	56	0.159	0.035	0.025	−0.014	0.084

**Table 5:** Descriptive statistics and chi-square analysis of clinical variables by mechanism of polytrauma.

		Car Crash		Fall		Other Mechanism		Simulation Exercise		Chi Square Test Sig.
		Count	Column Valid *n*%	Count	Column Valid *n*%	Count	Column Valid *n*%	Count	Column Valid *n*%	
Localization of the trauma	Head trauma	0	0	1	10	0	0	0	0	0.00
	Chest trauma	2	3.6	0	0	2	22.2	0	0	
	Abdomen trauma	1	1.8	0	0	0	0	0	0	
	Pelvis trauma	1	1.8	0	0	0	0	0	0	
	Lim injury	7	12.5	0	0	2	22.2	0	0	
	Association of areas affected	45	80.4	9	90	5	55.6	0	0	
	Simulation exercise	0	0	0	0	0	0	1	100	
Number of region affected	1 region affected	6	10.5	0	0	3	33.3	0	100	0.00
	2 regions affected	12	21.1	4	40	1	11.1	0	0	
	3 region affected	8	14	1	10	1	11.1	0	0	
	>3 region affected	31	54.4	5	50	4	44.4	0	0	
	Simulation exercise	0	0	0	0	0	0	1	100	
Sex	M	36	63.2	8	80	6	66.7	1	0	0.66
	F	21	36.8	2	20	3	33.3	0	0	
Oxygen	Mask	20	35.1	4	40	3	33.3	0	0	0.277
	Ambu Bag	6	10.5	0	0	0	0	0	0	
	No	15	26.3	2	20	6	66.7	1	100	
	Ventilator	13	22.8	2	20	0	0	0	0	
	Taken into in-tub	3	5.3	2	20	0	0	0	0	
Intubation	No	39	68.4	8	80	9	100	1	100	0.534
	With induction	14	24.6	2	20	0	0	0	0	
	Without induction	4	7	0	0	0	0	0	0	
External cardiac massage	No	55	96.5	10	100	9	100	1	100	0.888
	Yes	2	3.5	0	0	0	0	0	0	
Defibrillation	No	57	100	10	100	9	100	1	100	0.352
	Yes	0	0	0	0	0	0	0	0	
Extrication	No	54	94.7	9	90	7	77.8	1	100	
	Yes	3	5.3	1	10	2	22.2	0	0	

## Data Availability

Data is contained within the article. Data is unavailable due to privacy or ethical restrictions, a statement is still required.
